# A Dose Relationship Between Brain Functional Connectivity and Cumulative Head Impact Exposure in Collegiate Water Polo Players

**DOI:** 10.3389/fneur.2020.00218

**Published:** 2020-04-02

**Authors:** Derek C. Monroe, Nicholas J. Cecchi, Paul Gerges, Jenna Phreaner, James W. Hicks, Steven L. Small

**Affiliations:** ^1^Department of Neurology, University of California, Irvine, Irvine, CA, United States; ^2^Department of Ecology and Evolutionary Biology, University of California, Irvine, Irvine, CA, United States; ^3^Department of Psychological Science, University of California, Irvine, Irvine, CA, United States; ^4^School of Behavioral and Brain Sciences, University of Texas at Dallas, Dallas, TX, United States

**Keywords:** head impacts, concussion, sports, inhibitory control, brain connectivity, electroencephalography

## Abstract

A growing body of evidence suggests that chronic, sport-related head impact exposure can impair brain functional integration and brain structure and function. Evidence of a robust inverse relationship between the frequency and magnitude of repeated head impacts and disturbed brain network function is needed to strengthen an argument for causality. In pursuing such a relationship, we used cap-worn inertial sensors to measure the frequency and magnitude of head impacts sustained by eighteen intercollegiate water polo athletes monitored over a single season of play. Participants were evaluated before and after the season using computerized cognitive tests of inhibitory control and resting electroencephalography. Greater head impact exposure was associated with increased phase synchrony [*r*_(16)_ > 0.626, *p* < 0.03 corrected], global efficiency [*r*_(16)_ > 0.601, *p* < 0.04 corrected], and mean clustering coefficient [*r*_(16)_ > 0.625, *p* < 0.03 corrected] in the functional networks formed by slow-wave (delta, theta) oscillations. Head impact exposure was not associated with changes in performance on the inhibitory control tasks. However, those with the greatest impact exposure showed an association between changes in resting-state connectivity and a dissociation between performance on the tasks after the season [*r*_(16)_ = 0.481, *p* = 0.043] that could also be attributed to increased slow-wave synchrony [*F*_(4, 135)_ = 113.546, *p* < 0.001]. Collectively, our results suggest that athletes sustaining the greatest head impact exposure exhibited changes in whole-brain functional connectivity that were associated with altered information processing and inhibitory control.

## Introduction

Sport-related concussion, a form of mild traumatic brain injury (mTBI) induced by biomechanical forces, is a clinical diagnosis of abnormal brain function based on the presence of signs and symptoms without neuroimaging evidence of structural injury (1). The neurometabolic cascade associated with mTBI has been well-described from two decades of research (2), but neither this work nor more recent investigations employing electroencephalography (EEG) in humans have yet produced an objective measure that can confirm or refute the concussion diagnosis (3). The lack of a physiological definition of concussion to guide diagnostic and prognostic criteria has also contributed to growing concern for contact sport athletes who are likely to sustain repeated head impacts capable of producing brain injury, but who have neither symptoms nor professional evaluation.

Water polo is one such contact sport that carries a risk of head and face injury in international competition (4, 5). Recent survey data reveal that 36% of USA Water Polo members report sustaining at least one concussion during their playing tenure (6), a lifetime incidence that is comparable to that observed in soccer (7). However, the respondents also reported sustaining an average of two asymptomatic head impacts during a typical practice or game, a rate of exposure consistent with prospective data from *in vivo* monitoring over three competitive seasons (8). In some contact sports (e.g., American football, soccer, hockey) the accumulation of these impacts is believed to contribute to clinically significant neurological dysfunction years after exposure has ceased (9, 10). Though these injuries appear too subtle to be detected by cognitive testing after a single season of exposure, they become apparent when relating cognitive performance to neurobiological measures of injury and objective measures of head impact exposure (11).

Brain function arises from activity-based coupling across distributed neural networks that represent the brain's hierarchical (i.e., small-world) organization (12, 13), a balance between segregated and integrated information processing (14–16). Brain functional networks are altered after a concussion (17–19), an effect that can persist even after symptoms have abated (20). A few studies have observed similar changes after a single season of head impact exposure in football and rugby players, but these studies stratified their sample using a controversial method (21) of assessing injury thresholds based on head kinematic measures, (22) did not analyze individual differences in exposure, (23) and/or did not report objective measures of head impact exposure (24). These studies also used functional MRI methods that, despite offering useful insight into the pathophysiology of brain injury, are difficult to implement in prospective research designs and are not readily accessible to athletic training staff or even clinicians.

EEG represents a low-cost imaging method capable of measuring functional connectivity (FC) at fast time-scales not easily captured by MRI. Focal neural activity is governed by high-frequency oscillations (>20 Hz), whereas long-range, polysynaptic synchronization is instantiated in correlated slow-frequency oscillations (<7 Hz) (25, 26). Accordingly, fast-rhythm networks tend to be sparser and more clustered, and slow-rhythm networks tend to be denser with more synchronous activity (27). Affective and cognitive dysfunction across a range of neurological disorders has been attributed to disrupted brain network organization arising from aberrant synchronization in thalamocortical circuits (28, 29). Graph theoretic measures have gained popularity as a means to summarize quantitatively these organizational properties (i.e., density, clustering, efficiency) of large-scale brain networks, in health and disease (30, 31).

Several studies have used EEG to examine functional network properties in athletes after mTBI. Teel and colleagues observed increased slow-rhythm synchrony in recovered (i.e., asymptomatic) athletes post-concussion relative to healthy, non-concussed control athletes, but did not use graph measures to support their inferences about the meaning of these patterns for brain network organization (32). However, this pattern is consistent with reports of hyper-synchrony after mTBI using magnetoencephalography (33, 34) and is supported a recent review of 126 neuroimaging studies that concluded increased FC is a fundamental response to brain injury (35). In contrast, Cao and Slobounov observed that athletes diagnosed with a concussion exhibited decreased long-range connectivity, and increased local connectivity, seven days post-injury relative to non-injured athletes (36). Using graph theoretical measures, the authors interpreted these changes as a loss of network small-worldness and a shift toward network “randomness.” However, the control athletes in this study were all engaged in contact and collision sports (football, rugby, hockey) and thus had likely been exposed to repeated, asymptomatic head impacts, potentially confounding the interpretation of these group differences. Additionally, graph measures are frequency band-specific and can be influenced by the amplitude- and phase-dependence of the connectivity measures (37, 38). Neither were accounted for in this study.

To better understand the effects of repeated head impact exposure on brain FC, we monitored intercollegiate water polo athletes for head-impact frequency and magnitude during a season of competition. Specifically, we sought to test a dose relationship between head-impact exposure and changes in EEG-derived, whole-brain FC and small-world network characteristics. To provide context for the potential clinical significance of these effects, we used a multivariate modeling technique to characterize the relationship between changes in spontaneous brain activity and performance on computerized tests of inhibitory control. Then, we determined whether head impact exposure contributed to changes in this brain-behavior relationship.

## Methods

### Subjects

University students on the rosters of the Men's (*n* = 9) and Women's (*n* = 12) Club Water Polo teams participated by undergoing assessment both before and after a season of intercollegiate competition sanctioned by the Collegiate Water Polo Association. One male and two female athletes were excluded due to poor quality EEG recordings (>5 contaminated channels) at one or both sessions. Analysis was performed on data from the remaining eighteen participants who were 18–23 years old, right-handed, and reported no medication use. Two participants reported sustaining a concussion more than 12 months prior to the beginning of the season. All study procedures were approved by the Institutional Review Board of the University of California, Irvine. Written informed consent was obtained from all participants prior to assessment.

### Materials and Methods

#### Head Impact Monitoring

Participants were fitted with SIM-G inertial sensors, designed for both land-based sports and water polo, that relayed data to a sideline device (Triax Technologies; Norwalk, CT). To meet the sensor manufacturer's specifications and recommendations, each SIM-G sensor was inserted into a player's water polo cap that had been modified to include a Velcro pocket designed to couple the sensor with the occipital protuberance. Laboratory evaluations of the SIM-G demonstrate that it can record peak linear and rotational acceleration when coupled tightly to the occipital protuberance in a headband (39), and the sensor performed comparably when secured using a water polo cap (40). Impact data were collected across 22 games (11 men's games; 11 women's games).

The SIM-G sensors recorded the peak linear acceleration (PLA), peak rotational acceleration (PRA), and peak rotational velocity (PRV) associated with each head impact. Due to a built-in filter, only impacts registering a PLA >16 g were recorded. The sensors also feature a complex algorithm that aims to distinguish hit-related head movement from commonly occurring non-impact transients or false-positives (e.g., voluntary head movement, adjusting of headgear, sensor movements about the head) based on impact waveform topography. However, these algorithms have demonstrated poor reliability (40) and the filter was disregarded. Instead, six research staff members evaluated video recordings of all games to identify and eliminate false positives through a consensus process.

#### SIM-G Preprocessing

At the conclusion of each water polo season, visual inspection of video recordings from all games determined the validity of recorded accelerative events as head impacts. The consensus process worked as follows: Six undergraduate research assistants reviewed accelerative events on their own. Due to limitations in head impact sensor technology, reviewers only confirmed accelerative events as head impacts if an impact was visible on video, the athlete's cap was above the water, and the cap remained coupled to the athlete's head. Two research assistants were each asked to review games individually, and then at a consensus meeting, any hits in which there was not agreement between reviewers were then subjected to group review and discussion. Impact legitimacy was ultimately decided by group consensus. Each reviewer's independent scoring was kept and compared to the group consensus (>85.4% agreement). Accelerative events deemed as false positives (91.4%) were excluded from exposure calculations.

Due to the interdependence of the kinematic measures (PLA, PRA, PRV), and the known inaccuracies of inertial sensors in sport applications, all confirmed impacts were subjected to a Principal Component Analysis (PCA) to transform kinematic measures orthogonally into a new composite measure of relative impact severity in a procedure described by Greenwald et al. (41). Briefly, for each confirmed impact, the input biomechanical measures (PLA, PRA, PRV) were mean-centered and scaled by the variance of each measure (i.e., z-transformed), a principal component score (PCS) was calculated as the sum of each composite variable weighted according to the variance explained (i.e., the eigenvalues of the covariance matrix of the mean-centered and normalized data), and individual weighted cumulative head impact exposure (wCHI) was computed as the sum of all PCSs sustained by each athlete during the season. wCHI serves as a measure of the frequency and magnitude of head impacts sustained by each athlete.

#### Inhibitory Control

Inhibitory control was assessed using two interference tasks, Flanker and Stroop, and administered using OpenSesame (v3.1.9), an open source presentation program (42). Participants completed the tasks on a laptop computer with a full keyboard while seated at a desk in a sound-attenuated room.

##### Flanker interference task (FIT)

The Eriksen Flanker Interference Task (FIT) was used to test the effects of interfering stimuli on stimulus-response (43). The task consisted of 200 trials (ISI = 1,000 ms) consisting of five white arrows presented on a black background. one hundred trials contained congruent stimuli (< < < < <; >>>>>), and 100 trials contained incongruent (< < > < <; >> < >>) stimuli. Participants were instructed to press a key to indicate the direction of the middle arrow as quickly and as accurately as possible. Arrow selection and reaction time were recorded for offline processing. Trials with reaction times <30 ms and >3,000 ms were removed, and an average weighted reaction time (wRT) was computed for congruent and incongruent trials [(Reaction Time)^*^(1/Accuracy)^*^100]. FIT performance was operationalized as weighted reaction time interference score (wRTI): the difference in wRT between the congruent and incongruent trials. A higher (more positive) wRTI indicates worse performance and greater interference.

##### Stroop color-word interference task (SCWIT)

A computerized Stroop Color-Word Interference Task (SCWIT) was used to test distributed attentional processing (44). The task consisted of three blocks, each with 120 trials consisting of a color stimulus (red, green, blue, yellow) presented against a black background. Congruent stimuli were presented in blocks 1 (5 colored X's) and 2 (matching color-words). In block 3, the color and the color-word were incongruent. Participants were instructed to indicate the color of the stimulus by striking the appropriate key with the index finger of their dominant hand. Accuracy and reaction time were recorded for offline processing. Trials with reaction times <30 ms and >3,000 ms were removed, and an average weighted reaction time (wRT) was computed for each block [(Reaction Time)^*^(1/Accuracy)^*^100]. SCWIT performance was operationalized as weighted reaction time interference score (wRTI): the difference in wRT between the 1st and 3rd blocks. A higher (more positive) wRTI indicates worse performance and greater interference.

#### EEG Acquisition

EEGs were recorded using 32 Xpress Twist dry, active electrodes (actiCAP) arranged in 10–20 system and connected to a LiveAmp amplifier (Brain Products GMBH) connected via Bluetooth to a laptop computer. Scalp electrical activity was recorded for 5 min at a sampling rate of 500 samples *per second* (500 Hz) with respect to a common reference (Cz). Participants were seated in a sound attenuated chamber and asked to remain as still as possible and breathe normally with their eyes-closed while the EEG was recorded. The EEG was imported into MATLAB 2018b (Mathworks, Natick, MA) for offline pre-processing and analysis.

#### EEG Pre-processing and Analysis

EEG data were pre-processed and analyzed using functions from three toolboxes, artscreen (https://github.com/mdnunez/artscreenEEG), EEGLAB (45), and FieldTrip (46). Continuous EEG data were segmented into non-overlapping 2-s epochs (1,000 samples each) and linear trends were removed. The data were band pass filtered (1–50 Hz) using a high pass Butterworth filter (1 Hz pass band with a 1 dB ripple and a 0.25 Hz stop band with 10 dB attenuation) and a low pass Butterworth filter (50 Hz pass band with 1 dB ripple and a 60 Hz stop band with 10 dB attenuation). Filtered data were visually screened for channels and epochs containing excessive noise, and the resultant time series was subjected to an Infomax independent component analysis (45), from which components containing characteristic physiological artifacts (including eyeblinks, eye movements, and cardiac rhythms) were identified and removed (47). Remaining components were transformed back to channel space and four off-the-head (ridge) channels that were most often contaminated with artifact were removed from further analysis. Channels removed due to excessive noise were interpolated using spherical spline interpolation, and a surface Laplacian transform was applied as a reference-free technique to address confounding effects of signals recorded against a common reference (48, 49). To standardize the number of artifact-free epochs that were used for between subject comparisons global field power was calculated as the sum of squared amplitudes (μV^2^) across all channels for each epoch, and the 40 epochs (80 s; 40,000 samples) closest to the median for each subject were selected for further analysis.

##### Spectral power density (SPD)

A multitaper frequency transformation employing discrete prolate spheroidal sequences with a frequency smoothing of 3 Hz was applied to the pre-processed data to produce the power spectra from 1 to 50 Hz (0.5 Hz resolution) in each 2 second epoch (1,000 samples). Normalized spectral power density (SPD) was calculated from the power (μV^2^/Hz) at each frequency normalized to the average power across all frequencies within each channel. Average SPD(%) was computed by averaging across channels and frequencies for each of the five frequency bands of interest: delta (1–4 Hz), theta (4.5–7 Hz), alpha (7.5–15 Hz), beta (15.5–30 Hz), and low gamma (30.5–50 Hz). Pre- and post-season group average SPD are depicted in Supplemental Figure 1.

##### Functional connectivity

Standard practice suggests that multiple methods be used to compute FC from EEG data (48, 50). In this study, connectivity strength was estimated with coherence (COH), a regression method, and debiased weighted phase lag index (dWPLI), a phase synchronization method, computed between pairs of channels. COH describes the degree of correspondence between signals in phase and amplitude at one frequency, (50) whereas a value near 0 indicates a random difference and a value equal to 1 indicates no difference in phase and amplitude between channel pairs. COH estimates of connectivity can be inflated by volume conduction, an effect that can be mitigated by quantifying the degree of non-zero phase lag between two time series. The dWPLI was computed from the imaginary part of the cross-spectrum and debiased with respect to sample size (48, 51). A dWPLI value close to “1” indicates strong phase-lagged coupling while zero indicates an absence of coupling. The dWPLI is invariant to linear mixing of independent signals and the effects of volume conduction. COH and dWPLI were computed from the complex cross spectrum derived from the same multitaper transformation described above (1–50 Hz; 0.5 Hz steps).

To limit the potential for spurious FC, a null distribution of FC (dWPLI, COH) was estimated from surrogate data, which were generated by randomly dividing the amplitude time series (in each channel) and reversing the order of both halves (52). This process was repeated 1,000 times on each participant's resting-state EEG at each time point (pre-season, post-season). Observed synchronization (between pairs of channels at each frequency) was deemed to be statistically significant when larger than 95% of the surrogate data (computed between the same channels and at the same frequency). Finally, suprathreshold dWPLI and COH were averaged across channels and frequencies to obtain mean, whole-head FC for each of the five frequency bands of interest: delta (1–4 Hz), theta (4.5–7 Hz), alpha (7.5–15 Hz), beta (15.5–30 Hz), and low gamma (30.5–50 Hz).

##### Network graph measures

Graph theoretical analyses provide a framework for detecting changes in the topological properties of brain networks formed by nodes (channels) and their connecting edges, operationalized in this study by COH and dWPLI. Two “small-world” graph metrics were used: characteristic path length, calculated as the average fewest number of edges required to transfer between nodes, as an indicator of the serial information transfer, and average clustering coefficient, calculated as the average ratio of existing connections among the node's neighbors and all possible connections, as an indicator of local interconnectivity (53). Betweenness centrality was calculated as the fraction of shortest paths that travel through a given node. Because path length varies inversely with efficiency, the node with highest betweenness centrality is by definition associated with the largest number of shortest paths and contributes greatly to network efficiency. Therefore, the maximum betweenness centrality in the network was used as measure of its central organization. Global efficiency was calculated as the average inverse of the shortest path length, representing a measure of the capacity for parallel information transmission over the network. All calculations were carried out using functions adapted from the Brain Connectivity Toolbox (31).

#### Statistical Analyses

As a manipulation check, to confirm that incongruent trials were successfully testing inhibitory control, three separate repeated 2 time × 2 task × 2 trial measures ANOVAS, with time (pre-season, post-season), task (FIT, SCWIT), and trial (Congruent, Incongruent) as repeated measures, were used to test whether accuracy, reaction time, and wRT differed between trials. Corrections for sphericity (Huynh–Feldt epsilon, ε) and partial eta-squared (η^2^) effect sizes are reported where necessary. Simple interaction effects were decomposed with tests for simple main effects.

Pearson correlation coefficients were computed to test our primary hypothesis of a dose-response relationship between head impact exposure (wCHI) and changes (Δ = post-season–pre-season) in EEG metrics (ΔdWPLI, ΔCOH, ΔSPD) at each frequency band. A sample size of 15 was deemed necessary to detect a large effect (|r| > 0.70) of wCHI on changes in brain dynamics (α = 0.05, β = 0.80) (i.e., at least half of the variance in FC can be attributed to wCHI) (54). We sought to detect large effects, rather than subtle ones, because of the poor stability and high interindividual variability observed in graph theoretic measures derived using EEG (37). Cao and Slobounov also reported large differences (0.95 < *Cohen's d* < 1.7) in small-world measures between healthy and concussed contact and collision athletes during the subacute recovery phase (36). Bonferroni corrections for multiple comparisons across frequency bands were applied (*p*-value^*^5); corrected *p*-values are reported. Correlation coefficients were bootstrapped (500 samples) and confidence intervals are reported to support effect size interpretation. All correlational analyses and ANOVAs were performed in SPSS 26 (IBM; Armonk, NY).

To test our hypothesis that head impact exposure alters the brain-behavior relationship, a series of behavior partial least squares (PLS) correlation analyses were performed using a publicly available MATLAB toolbox (http://www.rotman-baycrest.on.ca/pls, Version 6.1311050) (55). PLS is a multivariate statistical method that employs permutation tests to compute statistical significance, thus minimizing the risks associated with multiple comparisons, and bootstrap resampling tests to quantify coefficient reliability. For these reasons PLS is well-suited to handle our variables (SPD, dWPLI, COH) that span many frequencies and channels. For each PLS analysis, a “brain” data matrix consisted of columns representing (i) dWPLI computed from 1 to 50 Hz (0.5 Hz increments) between each pair of channels (37422 columns), (ii) COH computed from 1–50 Hz (0.5 Hz increments) between each pair of channels (37422 columns), and (iii) SPD computed from 1–50 Hz (0.5 Hz increments) at each channel (2,772 columns) for each subject at pre- and post-season (36 rows). A “behavior” matrix consisted of weighted interference scores (wRTI) from the SCWIT and FIT (2 columns) for each participant at both time points (36 rows). Finally, a matrix of orthogonal contrast weights tested the hypothesis that the patterns of spontaneous brain activity would change in a way that was associated with poorer performance on the interference tasks (i.e., increased wRTI) from pre- to post-season.

By performing a behavioral PLS correlation analysis, a singular value decomposition is used to compute an optimal fit between the “brain” and “behavior” matrices resulting in four latent variables (LV), one for each behavior at each time point. The statistical significance of each LV was tested using a permutation test (1,000 permutations), and significant LVs are identified as those for which fewer than 50 permutations (<5%) resulted in a singular value greater than what was observed. Each LV represents a correlation, between brain and behavioral saliences, for which the reliability is estimated and 95% confidence intervals were computed using bootstrap resampling (500 bootstraps), and the associated singular value represents what percentage of the covariance that can be attributed to that LV. Scalp scores (similar to PCA component scores) are the summed product of each participant's brain matrix with the singular value and indicate how strongly that participant expresses the brain-behavior pattern indicated by the LV. For significant LVs, a Pearson correlation coefficient was computed to test the hypothesis that there was an association between wCHI and changes in scalp scores from pre- to post-season, and thus the degree to which changes in the brain-behavior relationship could be attributed head impact exposure.

To visualize the pattern of brain activity associated with changes in behavior, each bootstrapped mean salience was divided by its estimated standard error to obtain a normalized estimate of robustness, essentially a z-score given that the data are normally distributed. Saliences that were found to have a bootstrap ratio >1.96 (a 95% confidence interval) were interpreted as reliably and positively contributing to the observed brain-behavior relationship, whereas saliences with a bootstrap ratio <1.96 were interpreted as reliably and negatively contributing to the observed brain-behavior relationship. Resulting channel-wise z-maps were inspected to describe these patterns. A one-way ANOVA was used to determine whether average bootstrap ratios (after thresholding) were different across frequency bands.

The authors are aware of the well-documented inaccuracies of head impact kinematics measured using head-worn inertial sensors (40). Therefore, we used the same analytical approach described above to relate changes in FC to the number of confirmed head impacts (nHI) each athlete sustained, without considering the magnitude of those impacts (see Supplemental Text 1).

## Results

### Head Impact Exposure

**Figure 1:**
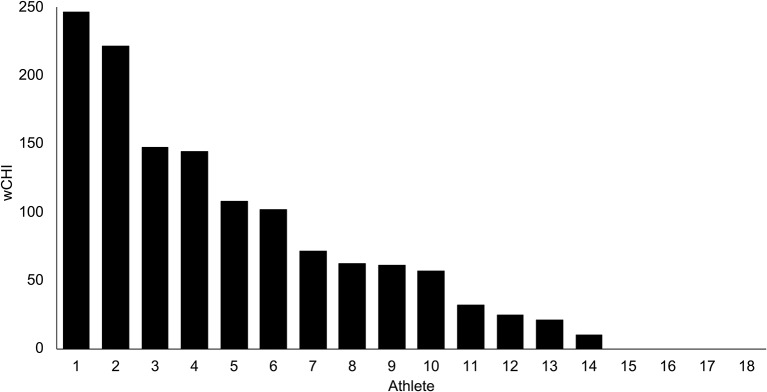
Weighted cumulative head impact exposure (wCHI) measured in 18 intercollegiate water polo athletes across 11 games. wCHI is a unitless composite measure: the sum of principal component scores which represent the normalized kinematics registered for each confirmed head impact.

18 athletes sustained 78 head impacts over 11 games [means (range): PLA = 36.4 (16.2–94.1) g; PRV = 16.3 (0.8–43.9) rad/sec; PRA = 4.4 (0.2–17.40) krads/sec^2^]. The distribution of weighted cumulative head impact exposure (wCHI) values derived from these kinematic parameters is depicted in Figure 1.

### Performance on Flanker and Stroop Color Word Interference Tasks

There was a task by trial interaction effect on task performance based on reaction time [*F*_(1, 17)_ = 18.322, *p* = 0.001, ε = 1.00, η^2^ = 0.519], accuracy [*F*_(1, 17)_ = 5.718, *p* = 0.029, ε = 1.00, η^2^ = 0.252], and wRT [*F*_(1, 17)_ = 16.617, *p* = 0.001, η^2^ = 0.494] (Table 1). Simple main effects revealed that reaction times and wRT were slower on incongruent trials compared to congruent trials (*p* < 0.001) and on the Stroop (SCWIT) compared to the Flanker (FIT) (*p* < 0.001). Accuracy was worse on incongruent trials relative to congruent trials (*p* < 0.001), but did not differ between tasks (*p* = 0.316).

**Table 1:** Task performance by trial, before and after the season expressed as group averages and standard deviations.

		**Flanker inhibitory task**				**Stroop interference task**			
**Time**	**Trial type**	**ACC** **(%)**	**RT** **(ms)**	**wRT**	**wRTI**	**ACC** **(%)**	**RT** **(ms)**	**wRT**	**wRTI**
Pre-season	Congruent	99.33 1.14	440.30 66.99	443.54 69.35	56.88 31.50	97.69 1.75	702.96 83.75	719.40 82.69	154.25 138.55
	Incongruent	95.67 3.73	478.84 66.67	500.42 64.51		96.16 3.12	837.87 126.40	873.64 144.40	
Post-season	Congruent	98.72 3.46	420.67 82.94	426.57 84.44	42.98 25.91	97.87 2.03	644.10 87.45	658.30 89.10	166.64 101.37
	Incongruent	96.28 5.10	451.54 66.56	469.55 66.83		96.34 3.34	794.58 162.36	824.94 165.01	

*ACC, Accuracy; RT, Response time; wRT, weighted response time; wRTI, weighted response time index*.

wCHI was not associated with ΔwRTI on the SCWIT (*r* = −0.284, *p* = 0.239) or FIT (*r* = 0.073, *p* = 0.767) tasks.

### Functional Connectivity, Network Measures, and Spectral Power Density

**Figure 2:**
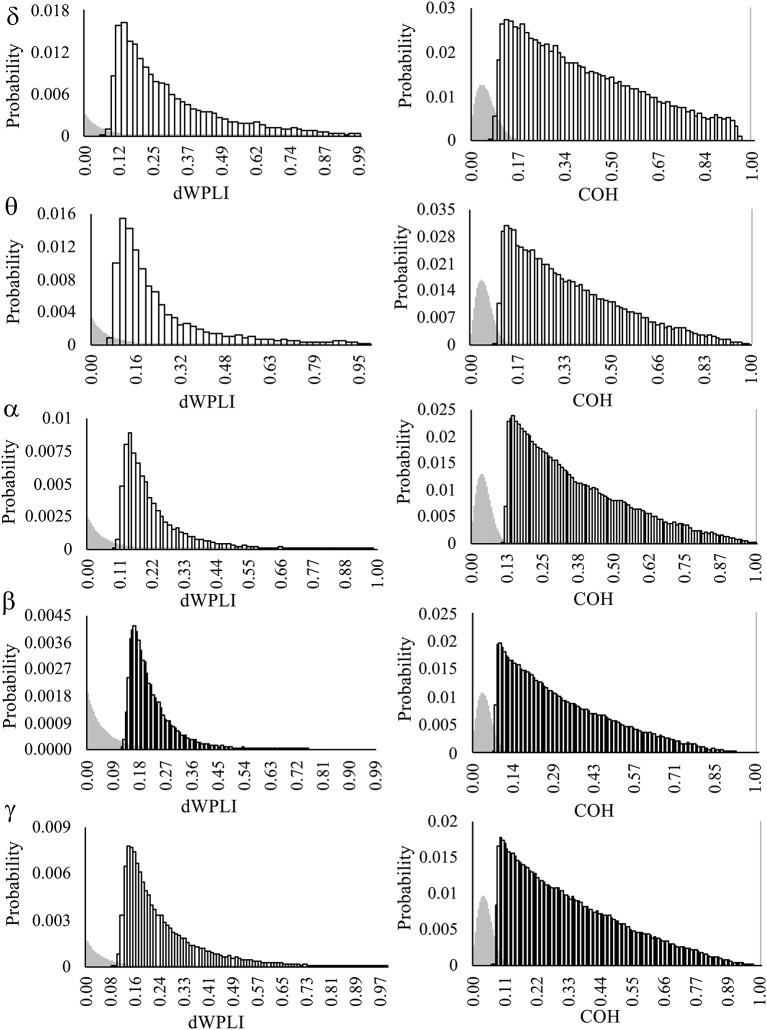
The probability distributions of connection weights (dWPLI, COH) from null distributions (gray) and observed distributions (white boxes). Observed distributions are comprised of FC greater than the top 5% of re-shuffled, surrogate data. Note the bimodal distribution of surrogate data from COH FC, which are most likely to exhibit near perfect coherence (>0.99; gray bar on the far right). Frequency bands: delta (δ = 1–4 Hz), theta (θ = 4.5–7 Hz), alpha (α = 7.5–15 Hz), beta (β = 15.5–30 Hz), and low gamma (γ = 30.5–50 Hz).

Bimodal null distributions of FC as estimated by coherence (COH), with predominant peaks >0.99, were observed across all frequency bands (Figure 2). This represents a positive bias in the surrogate data that prevented accurate thresholding of observed COH. Therefore, all analyses of FC were restricted to the debiased weighted phase lag index (dWPLI).

**Figure 3:**
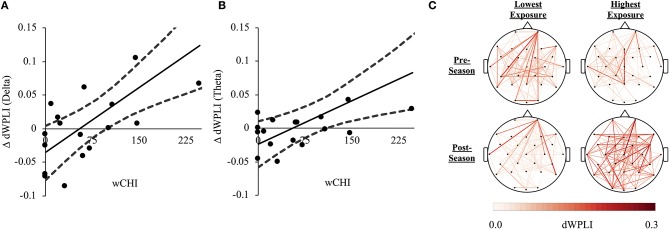
**(A)** A direct relationship between wCHI and change in FC (ΔdWPLI) from pre-season to post-season measured in the delta frequency band (1–4 Hz). Athletes sustaining the greatest wCHI exhibited the greatest increases in delta band dWPLI. **(B)** A direct relationship between wCHI and ΔdWPLI measured in the theta frequency band (4.5–7 Hz). Athletes sustaining the greatest wCHI exhibited the greatest increases in theta band dWPLI. **(C)** Network edges formed by slow-rhythm (1–7 Hz) dWPLI were averaged across athletes in the lowest (Athletes 13–18; left column) and the highest tertile of wCHI (Athletes 1–6; right column), at pre-season (top row) and post-season (bottom row). The nodes (channels) are oriented such that the front of the head is at the top of the figure. The colorbar represents dWPLI (0.00-0.30). For illustrative purposes, each graph was thresholded to only show the strongest 30% of all group-averaged FC. The athletes sustaining the most wCHI exhibited increased connectivity in the slow-rhythm, whole-brain network relative to athletes sustaining no (or very little) wCHI.

wCHI was directly associated with changes in dWPLI (ΔdWPLI) in the delta [*r*_(16)_ = 0.716, *p* = 0.004, 95%CI(0.403, 0.881)] and theta [*r*_(16)_ = 0.626, *p* = 0.025, 95%CI(0.091, 0.875)] bands (Figure 3). There was no association between wCHI and ΔdWPLI in other frequency bands (|r| < 0.416, *p* > 0.430).

wCHI was directly associated with changes in global efficiency in delta [*r*_(16)_ = 0.673, *p* = 0.010, 95%CI(0.313, 0.861)] and theta [*r*_(16)_ = 0.601, *p* = 0.040, 95%CI(0.148, 0.835)] bands and clustering coefficient in delta [*r*_(16)_ = 0.720, *p* = 0.004, 95%CI(0.390, 0.890)] and theta [*r*_(16)_ = 0.625, *p* = 0.030, 95%CI(0.206, 0.847)] bands in dWPLI-derived networks. wCHI was not associated with changes in average characteristic path length or maximum betweenness centrality in any frequency band (|r| < 0.561, *p* > 0.075).

wCHI was not associated with ΔSPD in any frequency band (|r| < -0.471, *p* > 0.240).

### Partial Least Squares (PLS) Correlation Between Brain and Behavior

**Figure 4:**
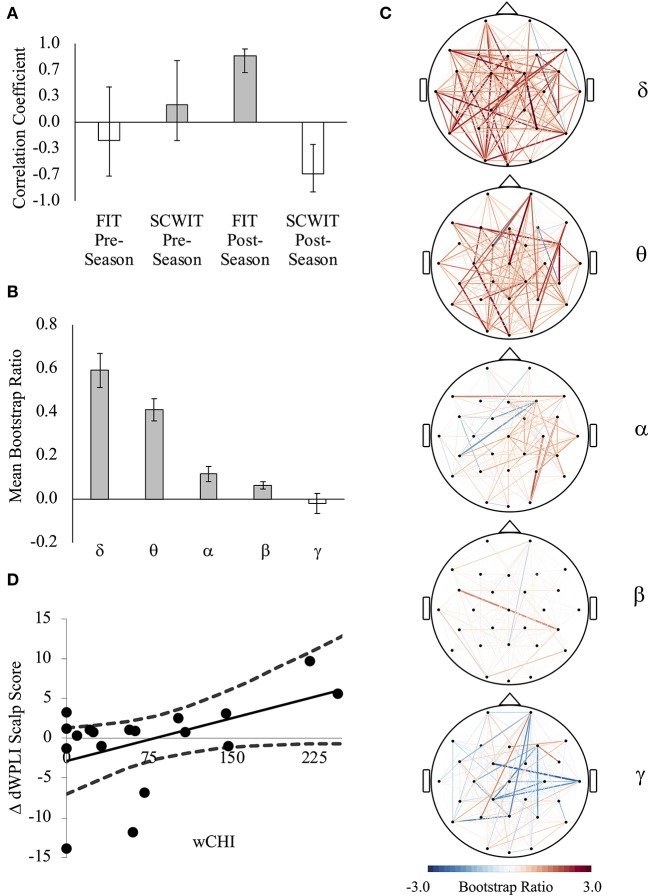
**(A)** The first latent variable reveals a pattern of brain connectivity that was strongly directly correlated with Flanker interference task (FIT) weighted reaction time index (wRTI) and inversely correlated with Stroop color-word interference task (SCWIT) wRTI at post-season. (Lower wRTI = better performance, less interference). **(B)** An individual's scalp score is a measure of how strongly their brain-behavior data represent the pattern indicated by the latent variable **(A)**. There was a direct relationship between wCHI and change (Δ) in scalp scores indicating that a pattern of network connectivity that was associated with worse FIT performance and better SCWIT performance was exhibited more by athletes sustaining high wCHI and less by athletes sustaining low wCHI. **(C)** FC that contributed the most positively (red) and most negatively (blue) to the observed relationship **(A)**. The color of each line represents the average of all significant bootstrap ratios (>1.96 or <1.96) for that connection across each frequency band. **(D)** There was a linear quadratic trend for the average of all significant bootstrap ratios (>1.96 or <1.96) in each frequency band [*F*_(1, 135)_ = 23.420, *p* < 0.001], indicating that dWPLI in delta and theta bands contributed the most positively.

In comparing brain FC (dWPLI) and inhibitory control, one latent variable was significant (*p* = 0.021) and explained 41% of the covariance in the brain-behavior relationship. This represented a pattern of synchrony that was directly associated with high interference on the FIT and inversely associated with interference on the SCWIT after the season (Figure 4A). On average (across all channels), there was a difference between the relative contribution of FC to this relationship across frequency bands [*F*_(4, 135)_ = 113.546, *p* < 0.001]. A significant quadratic trend [*F*_(1, 135)_ = 23.420, *p* < 0.001] indicated that this pattern could be attributed to greater dWPLI at frequencies < 7 Hz (delta, theta) (Figures 4B,C). At the individual level, wCHI was positively associated with changes in scalp scores across the season [*r*_(16)_ = 0.481, *p* = 0.043, 95%CI(0.054, 0.736)] (Figure 4D), indicating that athletes sustaining the most head impact exposure exhibited this pattern to a greater degree than athletes with less head impact exposure.

Similar results were obtained using only impact frequency data (see Supplemental Text 1).

## Discussion

To our knowledge, this is the first report of a linear dose relationship between changes in brain functional connectivity (FC) and the frequency and magnitude of repeated, asymptomatic sport-related head impacts. Our finding that repeated head impact exposure increases FC in slow frequency bands across the whole brain supports an existing body of neuroimaging data showing that neurological disturbances are commonly accompanied by increased functional connectivity (35). Using functional MRI, altered FC within default-mode and salience networks has also been observed during acute (<36 h) (56) and subacute (10 days-4 weeks) (19, 57) recovery after mTBI (i.e., symptomatic head impacts), after blast-induced mTBI (58), and during long-term recovery (3–6 months) after moderate and severe TBI (59). The clinical implications of these measures for behavior in otherwise asymptomatic athletes have proved elusive.

To that end, we observed a relationship between brain FC and dissociated performance on the Flanker task (FIT) and Stroop task (SCWIT) after the season. Due to differences in the way these tasks were designed and administered, we interpret this pattern as representing a differential effect of head impact exposure on so-called “reactive” and “proactive” strategies underlying cognitive control (60), as represented by performance on the FIT and SCWIT, respectively. Reactive control processes are recruited transiently to resolve conflict, only after presentation of the relevant stimulus (e.g., incongruent flanking arrows), while proactive strategies require sustained attention prior to the presentation of goal-relevant stimuli (e.g., a mismatch between the color of the word and the word itself). Proactive cognitive control can contribute to faster reaction times and better accuracy on interference tests, but comes with a metabolic cost, due to greater sustained network activity, and a reduced flexibility to accommodate other information or capacity to maintain other goals (61).

The FIT is commonly administered by randomly shuffling congruent and incongruent stimuli on a trial-by-trial basis. Although both proactive and reactive strategies are required, stimulus-response on this type of task is likely more contingent on reactive control processes as compared to the SCWIT (62). Our data reveal a pattern of FC, primarily driven by greater dWPLI in slow frequency bands, that was associated with poorer performance on the FIT and possibly impaired reactive control. This pattern was partially explained by accumulated repetitive head impacts, which is consistent with observations of aberrant brain structure and function associated with impaired reactive cognitive control across recovery milestones after mTBI (63–65). If asymptomatic head impacts impair cognitive control acutely—in the minutes following the impact—it is possible that athlete speed, reaction time, and decision making are affected, potentially increasing the risk of mTBI. This could explain why high school and collegiate football players are observed to sustain a greater dose (frequency and magnitude) of head impacts on days in which they experienced a symptomatic impact (i.e., concussion, mTBI) compared to days in which they did not (66).

We administered the SCWIT in a traditional “block” design, wherein participants were instructed to anticipate congruent or incongruent trials, thus deemphasizing the reliance on reactive control relative to the FIT. Stroop performance has been associated with FC in proactive control brain networks at rest (67). Likewise, the positive associations we observed between impact exposure and global efficiency and clustering coefficients in slow-rhythm, dWPLI-derived networks can be interpreted as representing high local and global integration necessary to meet the demands of proactive control during the SCWIT. Similar topological abnormalities have also been observed after moderate and severe brain injuries (68, 69), and our findings extend the thesis that increased FC following symptomatic head impacts is associated with a decentralization of, and attenuated capacity for, information processing (59, 70, 71) to include repetitive, asymptomatic impacts sustained during a single water polo season. On the other hand, higher cardiorespiratory fitness and greater physical activity have also been associated with behavioral and neuroelectric indices of proactive control processes across the lifespan (72, 73). Thus, we cannot rule out the possibility that this brain-behavior relationship is due to the accumulated exercise training across the season at the group level. Future studies that monitor a control group of athletes with orthopedic injuries, athletes competing in limited-contact sports, and/or non-athletes could further tease out the potential benefits of training from the detrimental effects of head impact exposure.

We note that Athlete 1, who sustained the greatest dose of exposure (Figure 1), exhibited a confounding deviation from the strong linear relationships between exposure and brain FC. We hypothesize that the reason for this finding was that Athlete 1 had a protracted recovery period between the end of the season and delayed post-season data collection (2 weeks later than the other participants). This would suggest that the changes in brain FC we observed in this sample are transient and may recover within weeks after abatement of impact exposure. Though considerable research has been conducted with the aim of quantifying the natural history of sport-related concussion to inform return-to-play protocols, relatively little is known about the recovery time course of brain FC from asymptomatic head impacts. Future studies that span multiple seasons could help quantify the degree to which patterns of FC are attributable to individual traits or to states that are sensitive to repeated head impacts.

The implications of our findings for contact-sport athletes at risk for repeated head impacts are not without limitations. First, it is well-known that head-worn inertial sensors exhibit poor accuracy for detecting “true” (vs. false-positive) head impacts (74). To address this limitation athletes were assigned to a single sensor and the data were subjected to a stringent collection and review process, resulting in only 8.6% of recorded events meeting inclusion criteria and factoring into the exposure (wCHI) calculation. These methods prevented us from quantifying exposure during practices, as caps (and therefore sensors) are not worn by the same athlete for the duration of all practices. These missing data might be particularly detrimental to estimating impact exposure sustained by goalies, the only position to report sustaining head impacts more frequently in practice than in games (6). It is important to note that the patterns observed with wCHI as an outcome measure were indistinguishable statistically from those incorporating only the frequency of head impacts (i.e., without consideration of the magnitude of those impacts), a relationship which could also be attributed to poor accuracy and reliability of the SIM-G sensors in estimating impact magnitude (40).

Second, we did not design this study, the first of water polo head impacts, with the intent of comparing the effects of repeated head impacts between male and female athletes. In some sports, women are at greater risk for sustaining a sport-related concussion than men (75) and experience different concussion-related symptoms than men (76). It is possible that these patterns are associated with sex-related differences in the physiological effects of cumulative head impact burden, an important topic worthy of future exploration in larger samples. Third, we observed a relatively low dose of exposure relative to other collegiate athletes engaged in contact and collision sports (e.g., football, soccer, lacrosse, rugby, ice hockey), and it is not known the degree to which these findings might generalize to athletes who sustain more head impacts of greater magnitude over a comparable number of games.

Notwithstanding these limitations, our results support a growing body of evidence that brain function can be altered by a single season of exposure to repeated, sport-related head impacts. The mixed findings from previous studies of coherence-estimated FC after mTBI may be due to the susceptibility of this measure to inflation by volume conduction of extracerebral electrical potentials (51). Consistent with this hypothesis we observed a large proportion of near perfectly coherent (COH > 0.99) activity in the surrogate data. Therefore, the novel observations we report here are based only on dWPLI, a measure of FC invariant to these effects. Similar patterns of increased neural synchrony have been associated with axonal injuries measured using diffusion weighted imaging (77, 78), and putative blood-based biomarkers of microstructural damage have demonstrated sensitivity to naturally occurring (79) and experimentally induced (80) head-to-ball impacts in soccer. Thus, we speculate that the impacts we observed were capable of causing deafferentation of cortical gray matter, propagating a neurometabolic cascade, inducing thalamocortical dysrhythmias (28), and ultimately increasing large-scale slow-rhythm synchrony we observed in the athletes sustaining the most exposure. These injuries would have plausibly translated to decreased spectral power density (SPD) and decreased local fast-rhythm FC, as measured after mTBI (34, 81), but it is possible that the subtle responses to asymptomatic impacts are focal to the impact site, and not as diffuse as after mTBI. By averaging over all channels, and examining only changes over the whole head or network, we may not have been able to detect these changes. It would be worthwhile for future studies to measure location-specific head-impact exposure (e.g., left vs. right; front vs. back) and perform analyses that consider location as a moderating factor.

## Conclusion

We report that the frequency and magnitude of head impacts sustained during a season of water polo competition were strongly associated with changes in whole-brain functional connectivity, particularly a pattern of slow-wave synchrony associated with a loss of inhibitory control. These findings support of a growing body of evidence that cumulative, sport-related head impacts can alter brain function, even in the absence of overt symptoms. This is the first study to employ water polo in the “sport as laboratory” assessment model, and our findings reinforce the need for more effective athlete monitoring protocols in contact sports.

## Data Availability Statement

The datasets generated for this study are available on request to the corresponding author.

## Ethics Statement

The studies involving human participants were reviewed and approved by Institutional Review Board of the University of California, Irvine. The patients/participants provided their written informed consent to participate in this study.

## Author Contributions

DM, NC, JH, and SS contributed to the conception and design of the study and interpretation of the results. DM, NC, PG, and JP contributed to data acquisition and analysis. DM wrote the first draft of the manuscript. NC and PG wrote sections of the manuscript. All authors contributed to manuscript revision, read, and approved the submitted version.

### Conflict of Interest

NC is founder and CEO of Counter, Inc., a company that manufactures and sells water polo equipment including protective headgear. The remaining authors declare that the research was conducted in the absence of any commercial or financial relationships that could be construed as a potential conflict of interest.
