# Supplementary material for: A Dose Relationship Between Brain Functional Connectivity and Cumulative Head Impact Exposure in Collegiate Water Polo Players
Source: Front Neurol. 2020 Apr 2;11:218. doi: 10.3389/fneur.2020.00218 (PMC7145392; doi:10.3389/fneur.2020.00218)
Supplement: Supplementary file 1 [file Table_1.DOCX]

**Supplemental Results**

*Functional Connectivity & Network Measures*

Head impact frequency (nHI) was directly associated with changes in the debiased weighted phase lag index (ΔdWPLI) in the delta [r(16)=.711, p=.005, 95%CI(.423, .890)] bands. There was no association between nHI and ΔdWPLI in other frequency bands (|r|<.589, p>.050).

nHI was directly associated with changes in global efficiency [r(16)=.657, p=.015, 95%CI(.363, .861)] and clustering coefficient [r(16)=.719, p=.005, 95%CI(.460, .902)] in the delta band in dWPLI-derived networks.

*Partial Least Squares (PLS) Correlation*

In comparing brain FC (dWPLI) and inhibitory control, one latent variable was significant (p =.021) and explained 41% of the covariance in the brain-behavior relationship. This represented a pattern of synchrony that was directly associated with high interference on the FIT and inversely associated with interference on the SCWIT after the season. On average (across all channels), there was a difference between the relative contribution of functional connections to this relationship across frequency bands [F(4,135) = 113.546, p<.001]. A significant quadratic trend [F(1,135) = 23.420, p<.001] indicated that this pattern could be attributed to greater dWPLI at frequencies < 7 Hz (delta, theta). At the individual level, nHI was positively associated with changes in scalp scores across the season [r(16)=.522, p=.026, 95%CI(.078, .771)], indicating that athletes sustaining the most head impacts exhibited this pattern to a greater degree than athletes with sustaining fewer head impacts.

**Supplemental Figure 1:** Group averaged relative spectral power density across frequency bands before and after the season. Error bars represent standard deviations.
